# Danggui Buxue Tang, a Chinese Herbal Decoction Containing Astragali Radix and Angelicae Sinensis Radix, Modulates Mitochondrial Bioenergetics in Cultured Cardiomyoblasts

**DOI:** 10.3389/fphar.2019.00614

**Published:** 2019-06-21

**Authors:** Kenneth K.L. Kwan, Yun Huang, Ka W. Leung, Tina T.X. Dong, Karl W.K. Tsim

**Affiliations:** ^1^Shenzhen Key Laboratory of Edible and Medicinal Bioresources, Shenzhen Research Institute, Shenzhen, China; ^2^Division of Life Science and Center for Chinese Medicine, The Hong Kong University of Science and Technology, Hong Kong, Hong Kong

**Keywords:** Astragalus, Angelica, anti-oxidative activity, Chinese medicine, extracellular flux analyzer, mitochondrial bioenergetics, oxygen consumption rate

## Abstract

Danggui Buxue Tang (DBT) is an ancient herbal mixture containing Astragali Radix and Angelicae Sinensis Radix, and which are commonly consumed for “qi-invigorating” (i.e., stimulating vital energy/energy metabolism) as traditional Chinese medicine (TCM). The pharmacological activities of DBT in anti-oxidation, estrogenic, hematopoietic, and immunogenic have been reported; however, the role of DBT in cellular energy metabolism has not been determined. Here, we employed an extracellular flux analyzer to evaluate the mitochondrial respiration of cultured H9C2 cardiomyoblasts in present of DBT. The herbal extract of DBT was qualified chemically for the major ingredients, i.e. astragaloside, calycosin, formononetin, Z-ligustilide, and ferulic acid. The anti-oxidant activities of DBT, as well as its major ingredients, were determined by Folin-Ciocalteu assay, 2,2-diphenyl-1-picrylhydrazyl (DPPH) radical scavenging assay, and protective effect in tert-butyl hydroperoxide (tBHP)-treated cultured cardiomyoblasts. In addition, a real-time oxygen consumption rate (OCR) in herbal extract-treated cultured cardiomyoblasts was revealed by using a Seahorse extracellular flux analyzer. In addition, the transcript expressions of peroxisome proliferator-activated receptor gamma coactivator 1-alpha (PCG-1α) and other genes relating to mitochondria biogenesis were determined in cardiomyoblasts under different herbal treatments. DBT possessed the strongest anti-oxidant activity and protective effects on the oxidatively stressed cardiomyoblasts. By revealing the OCR in mitochondria, the health state of cultured cardiomyoblasts under DBT was improved *via* increase of basal respiration, proton leak, non-mitochondria, and adenosine triphosphate (ATP) production. Furthermore, the transcriptional activities of genes responsible for mitochondrial biogenesis and DNA replication were stimulated by application of DBT in cultures.

## Introduction

Traditional Chinese medicine (TCM) has been used as medicine, or health supplement, in China over thousands of years (Lu and Lu, 2014). Historically, TCM herbal decoction is prepared by a unique methodology with specific combination of different herbs as a formula. Among thousands of herbal formulae, Danggui Buxue Tang (DBT) is an ancient Chinese herbal decoction having a simple combination of two herbs. DBT was first described in *Neiwaishang Bianhuo Lun* by Li Dongyuan in China in AD 1247. DBT contains two common herbs, Astragali Radix [AR; roots of *Astragalus membranaceus* (Fisch). Bunge or *A. membranaceus* (Fisch). Bunge var. *mongholicus* (Bunge) Hsiao] and Angelicae Sinensis Radix (ASR; roots of *Angelica sinensis* Oliv). at the weight ratio of 5 to 1 (Lin et al., 2017). DBT is a well-known herbal decoction in clinical treatment for women suffering from menopausal symptoms (Gong et al., 2016a). Until now, numerous components have been identified in DBT decoction, and astragaloside IV, calycosin, formononetin, Z-ligustilide, and ferulic acid are considered to be major and active ingredients (Zheng et al., 2011). According to TCM theory, DBT promotes “qi” (vital energy) enhancement and “blood” (body circulation) nourishment. DBT has been proposed to possess pharmacological properties both *in vivo* and *in vitro*, which includes: i) stimulating hematopoietic function (Yim et al., 2000), ii) exhibiting estrogenic property (Choi et al., 2011), iii) accelerating bone regeneration (Wang et al., 2015), iv) promoting immune system (Yang et al., 2014), v) protecting cardiovascular and pulmonary system (Mak et al., 2006), and vi) engaging formation of capillary and blood vessel (Gong et al., 2016a).

DBT and its chemical ingredients showed protective effects to different organs in various models of oxidative stress-related defects. For example, the antioxidant properties of ASR-derived ferulic acid (Kikuzaki et al., 2002) and AR-derived astragalosides and isoflavonoids were clearly shown in both *in vitro* and *in vivo* (Chen et al., 2011). In ferulic acid knock-out DBT, a significant decrease of anti-oxidative property was revealed, and which strongly suggested the role of ferulic acid in DBT (Gong et al., 2016b). To achieve maximal anti-oxidative functions, astragalosides from AR inhibited ROS generation, as induced by high glucose, in rat retinal capillary endothelial cells (Qiao et al., 2017). In addition, the flavonoids from AR, e.g. calycosin and formononetin, inhibited ROS-related lipid peroxidation (Toda and Shirataki, 1998).

Mitochondrial bioenergetics is a measurement of cellular energy status. In live cells, a Seahorse extracellular flux analyzer could be used to reveal the profiling of mitochondrial bioenergetics. These bioenergetics parameters are oxygen consumption rate (OCR), spare respiratory capacity (SRC), referring to ability of mitochondria to increase ATP to theoretical maximum (Dranka et al., 2010), and proton leakage, representing part of OCR pushing the substrate cycle of proton pumping and proton leak across inner mitochondrial membrane and not coupling to ATP production (Brand, 2005).

An important function of mitochondria is to enhance ATP production and oxidative phosphorylation for balance of bioenergy (Owen and Sunram-Lea, 2011). In addition, mitochondrial has a crucial role in cell death, signaling transmission, and energy homeostasis (Friedman and Nunnari, 2014). Thus, the malfunction of mitochondria is proposed to be the primary cause of energy decline, as well as onset of aging process, including neurodegenerative disease and diabetes (Sivitz and Yorek, 2010). Improving the profile of mitochondrial bioenergetics could be an approach to prevent cell damage (Wallacea et al., 1997). Thus, the application of TCM in maintaining health status of mitochondria should be considered. DBT is commonly consumed in daily life, and which has been claimed to be “qi-invigorating” (i.e., stimulating vital energy/energy metabolism), but the effect of DBT in mitochondrial bioenergetics is still a mystery. Here, a detail oxidative phosphorylation parameter in mitochondria, after application of DBT, or other herbal extracts, in cultured cardiomyoblasts were determined in live cell dynamically by fluorescence-based respirometer. In addition, the expression of mitochondrial genes, as well as the morphology of mitochondria, was determined here in cultured cardiomyoblasts.

## Materials and Methods

### Standardization of DBT Extracts

The preparation of herbal extracts, e.g., DBT and water extracts of ASR and AR, was described previously (Song et al., 2004). Roots of 3-year-old *A. membranaceus* var. *mongholicus* (AR) from Shanxi Province and 2-year-old *A. sinensis* roots (ASR) from Minxian of Gansu Province were used. The authentication of plant materials was identified by Dr. Tina Dong at Hong Kong University of Science and Technology (HKUST). The authentication of these herbs was according to Hong Kong Materia Medica Standards. The voucher specimens (voucher # 02-9-1 for ASR and voucher # 02-10-4 for AR) were stored in Centre for Chinese Medicine of HKUST. To prepare the herbal extract, DBT (exact amounts of AR and ASR in a weight ratio of 5:1), AR, and ASR were boiled in 8 vol of water (v/w) for 2 h, and the extraction was repeated twice. The extracts were dried by lyophilization and stored at −80°C. Qualification of chemical markers of DBT and water extracts of ASR and AR was performed on an Agilent HPLC 1200 series system (Agilent, Waldbronn, Germany), equipped with a degasser, a binary pump, an autosampler, a thermos-stated column compartment, and a diode array detector. The mixtures were filtered by a 0.22-µm filter before separated by an Agilent ZORBAX Eclipse XDB-C18 column (1.8 µm, 50 mm × 4.6 mm). The mobile phase composed of 0.1% formic acid in acetonitrile (A) and 0.1% formic acid in water (B) according to the following gradient program: 0–2 min, isocratic gradient 20–20% (A); 2–7 min, linear gradient 20–34% (A); 7–12 min, isocratic gradient 34–34% (A); 12–16 min, linear gradient 34–65% (A); and 16–18 min, linear grad 65–80% (A). A pre-balance period of 5 min was used between each run. The flow rate was set at 0.4 ml/min; the column temperature was 25°C; and the injection volume was 5 µl. To determine amount of chemical markers of DBT, ASR, and AR extracts, the effluent was introduced into MS for further analysis. Mass spectrometry was performed on an Agilent QQQ-MS/MS (6410A) equipped with an ESI ion source was operated in positive and negative ion. The dry gas temperature was 325°C; drying gas flow rate: 10 L/min; capillary voltage: 4,000 V; nebulizer pressure: 35 psi; and delta electro multiplier voltage: 400 V. For the multiple reaction monitoring (MS/MS) analysis, two transition pairs were chosen for acquisition in MRM mode for calycosin, formononetin, Z-ligustilide, ferulic acid, and astragalosides. The collision energy value and fragmentor voltage were optimized in advance to obtain the highest abundance.

### Cell Cultures

The rat embryonic cardiomyoblast, H9C2 cell line, was obtained from American Type Culture Collection (ATCC, Manassas, VA). H9C2 cell line was maintained in high-glucose content Dulbecco’s modified Eagle’s medium (DMEM) supplemented with 100 IU/ml penicillin, 100 µg/ml streptomycin, and 10% fetal bovine serum (FBS) at 37°C in a 5% CO_2 _water-saturated incubator. All culture reagents were purchased from Invitrogen Technologies (Carlsbad, CA).

### Cell Viability Assay

Cell viability was performed by 3-(4,5-dimethylthiazol-2-yl)-2,5-diphenyltetra-zolium bromide (MTT) assay. Cells were seeded in 96-well plates at a density of 5,000 cells/ml. After 24-h drug treatment, cells in each well were incubated with 50 µl MTT (2 mg/ml, Invitrogen) at a final concentration of 0.5 mg/ml for 3 h at 37°C. After removal of the solution, DMSO was used to re-suspend the purple precipitate inside the cells at room temperature, and the absorbance was detected at 570 nm. The cell viability was calculated as a percentage of absorbance; while the value of vehicle control was set as 100%.

### Folin-Ciocalteu Assay

To reveal the amount of total phenolic compound, 20 µl of each herb sample together with 40 µl 10% (v/v) Folin-Ciocalteu solution (Sigma-Aldrich, St Louis, MO) was added into 96-well microplate and shaken for 10 min at room temperature in dark. Then, 160 µl Na_2_CO_3_ (700 mM) was introduced into the well. The assay plates were shaken and incubated at room temperature without light exposure for 2 h before optical displacement (OD) value at 765 nm was measured. At the same time, gallic acid (Sigma-Aldrich) was used as the reference chemical marker, and total phenolic amount of each extract was referring amount of gallic acid.

### Radical Scavenging Assay



The free radical scavenging activity of herbs extracts was measured by 2,2-diphenyl-1-picrylhydrazyl (DPPH) colorimetric assay. In brief, 150 µl of 0.1 mM DPPH solution with 50 µl of each extract (0–9 mg/ml) was added into 96-well microplate for 10 min at room temperature. The color of DPPH solution was changed from purple to yellow, indicating free radical scavenging or redox reaction. The OD value at 517 nm was measured. DPPH radical scavenging capacity was calculated as inhabitation percentage based on the following equation: inhibition [(%) = (A_0_-A_1_)/A_0_] × 100, where A_0_ is the OD value of the vehicle control, and A_1_ is the OD value of each herbs sample aliquot. In this assay, gallic acid (0–100 µM) was served as a positive control.

### Oxidative Stress Assay

To investigate the ability of DBT against tert-butyl hydroperoxide (tBHP)-induced cytotoxicity of oxidative stress, the cells were cultured in 96-well plate. After drug treatment for 24 h, tBHP (400 µM) were added into the wells for 3 h before MTT at 37°C, MTT was dissolved in 1 × PBS at a final concentration of 0.5 mg/ml. After the solution was removed, the purple organic crystal inside the cells was re-suspended in dimethyl sulfoxide (DMSO) and then measured at 570 nm absorbance. Before performed this experiment. The amount of tBHP (Sigma-Aldrich) and positive control (vitamin C, 1 mM) were optimized in cultured H9C2 cells.

### Mitochondrial Bioenergetics Analysis

To determine mitochondrial bioenergetics of H9C2 cell, real-time oxygen consumption by mitochondria in live cells was monitored by a Seahorse Bioscience XFp extracellular flux analyzer (Agilent). When the cell was exposed to various stimuli through multiple mitochondrial specific inhibitors from injection ports, the rate of oxygen consumption (OCR) by mitochondria in live cells could be measured by the change of fluorescence intensity at Seahorse XFp sensor cartridge. The seeding density of H9C2 cell was optimized at 5,000 cells per well, and thus this density was utilized for the experiments. In addition, mitochondrial-specific inhibitors and uncouplers (Sigma-Aldrich) were used at 1 µM oligomycin (complex V inhibitor), 3 µM FCCP (mitochondrial oxidative phosphorylation uncoupler), and 1 µM rotenone/antimycin A (inhibitors of complex I and complex III, respectively) to elicit maximal effects on mitochondrial respiration. The fluorescence background noise and interference were normalized by background correction wells (the wells without cells). Cells were seeded on XFp cell mini-plates (Agilent) and treated with herbal extracts for 24 h. The sensor cartridge of XFp analyzer was hydrated in a 37°C non-CO_2_ water-saturated incubator a day before the experiment. During the sensor calibration, cells were incubated in 37°C non-CO_2_ incubator in 180 µl assay medium (XF base medium, 10 mM glucose, 1 mM pyruvate, and 2 mM L-glutamine, pH 7.4 at 37°C) for 1 h prior to assay. The plate was placed into calibrated XFp extracellular flux analyzer. The OCR had three cycles of reading: 2 min of mixing solution, 2 min of reagent incubation, and 2 min of fluorescing intensity measurements consisted in each measurement cycle. The OCR was normalized to amount of cellular protein/well and corrected for extra-mitochondrial O_2_ consumption. After Seahorse XFp assay, the cells were lysed with high salt lysis buffer (50 mM Tris-HCl, pH 8.0, 500 mM NaCl, 5 mM EDTA, and 1% Triton X-100), and the amount of total protein of intact cells was determined using Bradford reagent (Bio-Rad; Hercules, CA). The bovine serum albumin (BSA) was used for the standard curve for the determination of protein content. The OCR data were expressed as pmol/min/µg protein. Each complex of the mitochondrial respiratory chain was provided in bioenergetics profile. In brief, six parameters of mitochondrial function were calculated from bioenergetics profile: basal respiration, ATP production, proton leak, maximal respiration, spare respiration capacity, and non-mitochondrial respiration.

### Mitochondrial Membrane Potential Assay

H9C2 cell was seeded on 96-well black multiwall plate at 4,000 cell/ml with clear bottom and grown for 24 h. The cultures were washed with 1× PBS twice and loaded with 100 µl JC-1 dye (20 µM; Sigma-Aldrich) at 37°C for 10 min under 5% CO_2_ r (v/v) at 37°C. The cells were washed with PBS twice for staining and incubated with DBT, as well as extracts of ASR, AR, and their mixture. Carbonyl cyanide-p-trifluoromethoxy-phenylhydrazone (FCCP; 100 µM), a mitochondrial uncoupler, was used as a negative control in the measurement. The accumulation of JC-1 dye in mitochondria was quantified by red fluorescence intensity (excitation 527 nm and emission 590 nm), which was monitored every 1 min for up to 60 min at 37°C. The changes in mitochondrial membrane potential were expressed as the percentage of initial level of corresponding control.

### Real-Time Polymerase Chain Reaction

Cultured H9C2 cells were seeded in a 6-well plate at 4,000 cells/ml and treated with herbal extracts, or other reagents, for 24 h. Total RNA was isolated by RNAzol reagent from Molecular Research Center Inc. (Cincinnati, OH), followed by reverse transcription into cDNA according to the manufacturer’s instructions (Invitrogen). RT-PCR was performed by using Fast Start Universal SYBR Green Master (ROX) according to manufacturer’s instructions (Roche Diagnostics; Mannheim, Germany). The primers for rat PCG-1α gene were 5’-GGA GCA ATA AAG CAA AGA GCA-3’ and 5’-GTG TGA GGA GGG TCA TCG TT-3’; the primers for rat TFAM were 5’-CAG AGT TGT CAT TGG GAT TGG-3’ and 5’-TTC AGT GGG CAG AAG TCC AT-3’; the primers for rat NRF1 were 5’-TTG ATG GAC ACT TGG GTA GC-3’ and 5’-GCC AGA AGG ACT GAA AGC AG-3’; the primers for rat PLOG were 5’-CTC CTA CCT GCC TGT CAA CC-3’ and 5’-GCT CCA TCA GCG ACT TCT TC-3’; the primers for rat TOP1MT were 5’-CCA AGG TGT TTC GGA CCT AC-3’ and 5’-GTT TGC CCG GTT GTA AGC TA-3’; the primers for rat TWINKLE were 5’-GAG GAC AGG GAG GAG GTC TT-3’and 5’-TGG TAA GGC CAA ACA TCA CA-3’. The rat GAPDH RNA was used as internal control in all case, and its primer sequences were: 5-AAC GGA TTT GGC CGT ATT GG-3’ and 5-CTT CCC GTT CAG CTC TGG G-3’. The SYSB green signal was detected by LightCycler 480 Ⅱ real-time PCR system (Roche; Basel, Switzerland). The transcript levels were quantified by using ΔΔCt value method under LightCycler 480 software release 1.5.1.62 SP3 in which the values of EPO genes were normalized first by GAPDH mRNA in the same sample before comparison. The products of PCR were analyzed by gel electrophoresis, and the analysis of melting curve was for confirmation of amplification.

### MitoTracker Staining and Confocal Microscopy

Cardiomyoblasts were seeded in 6-well plates at a density of 4,000 cells/ml on a glass slide. The cell was treated with herbal extract for 24 h. Following the treatment, the treated cell was strained by MitoTracker FM red (Invitrogen) at concentration of 150 nM for 30 min. The MitoTracker FM red was removed and washed by PBS, three times. The 63× objective was used in a laser scanning confocal microscope (LSM7 DUO, Zeiss). Fluorescence intensity was measured by Zeiss software, and mitochondria parameters were measured by ImageJ (Dagda et al., 2009).

### Transmission Electron Microscopy

H9C2 cells were seeded in 6-well plates at a density of 4,000 cells/ml on 100-mm culture dish. After the drug treatment, H9C2 cells were trypsinized and were fixed in 2.5% glutaraldehyde for 24 h. Then, the fixed cells were firmed by 1% osmium tetroxide. After, the cells were dehydrated and embedded in durcupan. The embedded samples were cut into 60 nm by using a diamond knife and mounted on Cu-grids. The images were viewed by transmission electron microscope (Philips CM100). The mitochondria parameters were measured by ImageJ.

### Statistical Analysis

Tandem mass spectrometry data were processed using Agilent Mass Hunter workstation software version B.01.00. Principal component analysis (PCA) of the sample was conducted using SIMCA-P version 13.0 (Umetrics, Sweden). The mitochondrial bioenergetics profile was visualized on Wave Desktop 2.3.0. All data were expressed as mean ± standard error of the mean (SEM). Statistical tests were performed with Student’s t-test and Dunnett’s test (one-way analysis of variance with multiple comparisons, SPSS, version 13). Statistically, difference was classified as significant (*) where *p* < 0.05, more significant (**) where *p* < 0.01, and highly significant (***) where *p* < 0.001.

## Results

### Phenolic Compounds in DBT

In chemical standardization of herbal extracts, an optimized LC-MS method was used to determine the amounts of major ingredients within DBT, e.g. astragaloside IV, calycosin, formononetin, ferulic acid, and Z-ligustilide, as reported (Zheng et al., 2012; **Supplementary Figure 1A**). The LC profiles of those chosen markers in herbal extracts were revealed (**Supplementary Figure 1B**); while the amounts of those chemical markers were calibrated (**Table 1** and **Supplementary Table 1**). A well-standardized herbal decoction is a must as to ensure the repeatability of all experiments. A PCA analysis of marker contents in DBT was conducted. Two ranking PCs, PC1 and PC2, described ∼53.9% and ∼43.1% of the total variability, respectively, and which accounted for ∼96% of total variance. The score plot showed that DBT, ASR, AR, and ASR+AR (simply adding AR extract and ASR extract in 5:1 ratio) could be clarified into four distinct groups (**Supplementary Figure 1C**). The loading plots for PC1 versus PC2 indicated different role of each variable in the extracts (**Supplementary Figure 1D**).

**Table 1:** Quantitative assessment of five chemicals in DBT, AR, and ASR extracts.

Marker chemical	DBT (ng/mL)^a^	AR (ng/mL)^b^	ASR (ng/mL)^c^
Calycosin	3,051.91 ± 16.36^d^	1,140.59 ± 0.12	–
Formononetin	764.54 ± 0.423	224.45 ± 10.84	–
Ferulic acid	267.46 ± 24.9	–	1,086.42 ± 40.86
Z-ligustilide	4.98 ± 0.43	–	34.19 ± 1.26
Astragal side IV	737.79 ± 5.73	475.24 ± 1.31	–

a Amount of chemicals in 1 mg/ml of DBT, determined by LC-MS/MS.

b Amount of chemicals in 1 mg/ml AR extract, determined by LC-MS/MS.

c Amount of chemicals in 1 mg/ml ASR extract, determined by LC-MS/MS.

d Values are in mean ± SD, n = 3.

### Anti-Oxidative Effect of DBT

**Figure 1:**
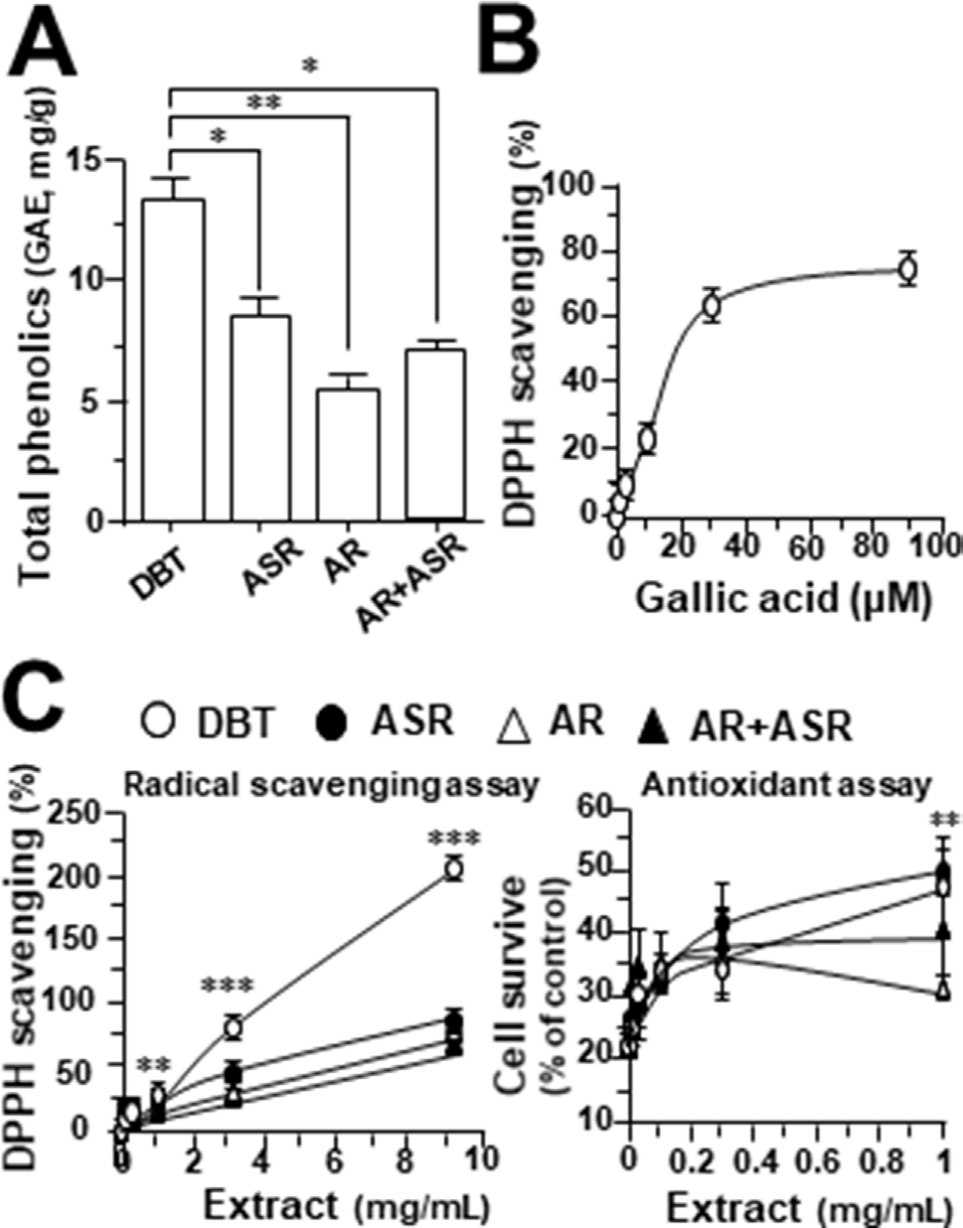
**(A)** Total amount of phenolic compounds in herbal extracts was measured using Folin-Ciocalteu assay, corresponding to equivalent amount of gallic acid (i.e., GAE in mg/g). **(B)** Radical scavenging ability was detected by colorimetric reaction in gallic acid. **(C)** Protection effect of herbal extract to H9C2 cells against oxidative stress, i.e. DPPH scavenging (left) and cell protection against 400 µM tBHP (right). The values are in percentage of control (no treatment) in mean ± SD, *n* = 4. Statistical difference was made of the sample with the lowest value of corresponding content or indicated, *p < 0.05, **p < 0.01, and ***p < 0.001.

To conduct a comprehensive analysis of anti-oxidative effects of herbal extracts of DBT, AR, ASR, and ASR+AR, free radical scavenging activity and total phenolic compounds were examined. Gallic acid was used as a reference marker, and total phenolic content of each herbal extract was represented by an equivalent amount of gallic acid. As shown in **Figure 1A**, DBT contained significant higher content of phenolic compounds, i.e., equivalent to ∼13 mg gallic acid/g of sample. The extracts of ASR, AR, and ASR+AR contained equivalent ∼8.5, ∼5, and ∼7 mg gallic acid/g, respectively. Moreover, the free radical scavenging activity was determined here, and gallic acid served as a control showing a dose-dependent manner (**Figure 1B**). At the same time, DBT showed significant higher, at least ∼2.5 folds, in DPPH scavenging activity, as compared to that of other herbal extracts (**Figure 1C**). Here, we proposed a correlation between free radical scavenging ability and amount of phenolic compounds in DBT might be closely related (**Figure 1A and C**). Thus, the anti-oxidative element within the herbal extract could be represented by total phenolic compounds.

H9C2 cell, a commonly used cardiomyoblast, was employed here in testing the role of DBT. A stress inducer, tBHP, was chosen to damage cultured cardiomyocytes in a dose-dependent manner (**Supplementary Figure 2A**). A sub-maximal induction of cell death at ∼400 μM was used for subsequent assay. The applied herbal extract by itself in cultures did not affect the cell viability (**Supplementary Figure 2B**). Application of herbal extracts of DBT, AR, ASR, and ASR+AR protected cardiomyocytes against oxidative insult in a dose-dependent manner (**Figure 1C**, right). Among the herbal extracts, DBT and ASR showed significant higher, at least ∼1.5 folds, in protecting cell from damage, as compared to that of AR and ASR+AR extracts at 1 mg/mL (**Figure 1C**, right).

### DBT Regulates Mitochondrial Bioenergetics

**Figure 2:**
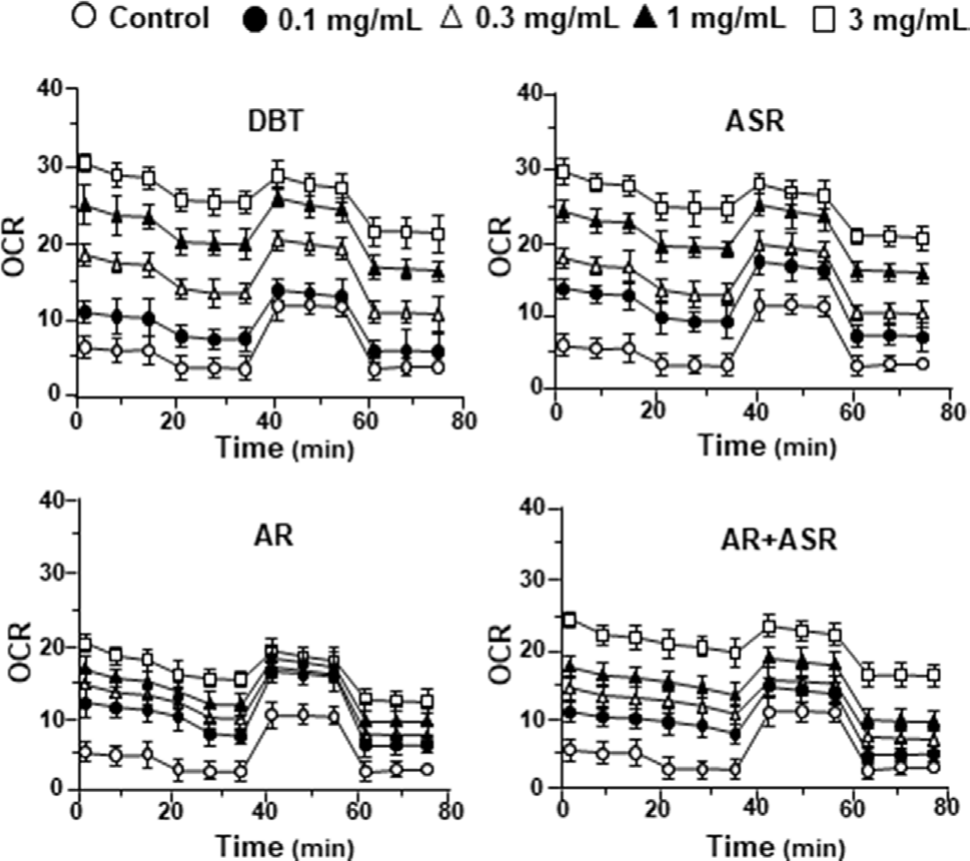
The extracts of DBT, ASR, AR, and ASR+AR modulate mitochondrial bioenergetics of H9C2 cells. Cultured H9C2 cells were treated with herbal extracts, as indicated, for 24 h before measuring oxygen consumption rate (OCR in pmol/min/mg of protein) with XFp Cell Mito Stress Test. The response of H9C2 cells after oligomycin (1 µM), FCCP (3 µM), and rotenone/antimycin A (1 µM) applied to the wells were recorded. Blank is the naive cell. Data are expressed in mean ± SD, *n* = 3, each with triplicate samples.

**Figure 3:**
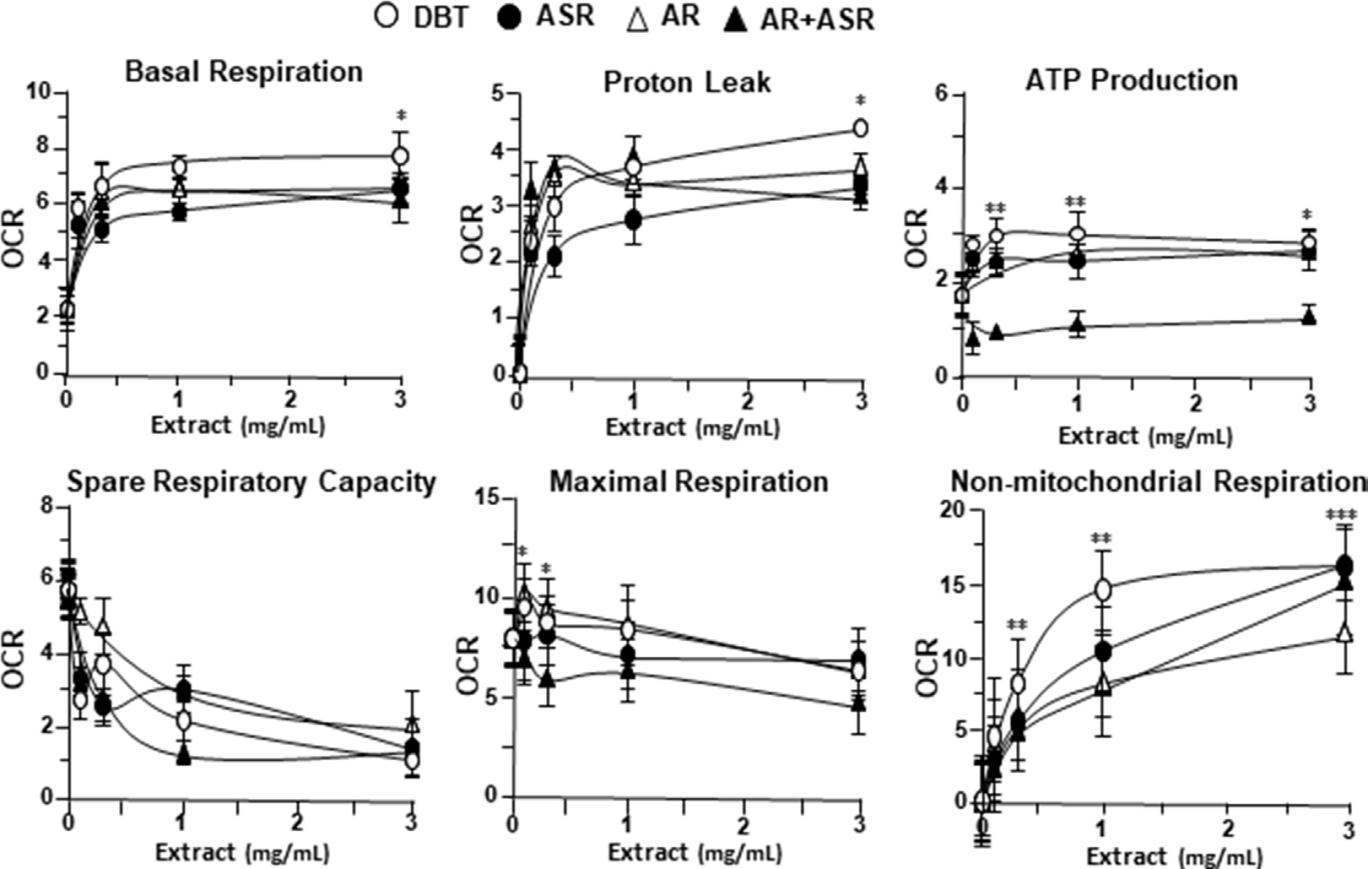
The parameters of mitochondrial respiration in DBT, ASR, AR, and ASR+AR applied H9C2 cells. Cultured H9C2 cells were treated with herbal extracts as that in **Figure 2**. The effects of different extracts to basal respiration, proton leak, ATP production, spare respiratory capacity, maximal respiration, and non-mitochondrial respiration were measured and compared. The OCR value in pmol/min/mg of protein was shown. Data are expressed as mean ± SD, *n* = 3, each with triplicate samples. Statistical comparison was made of the sample with the lowest value of corresponding concentration, *p < 0.05, **p < 0.01, and ***p < 0.001.

By using Seahorse XFp extracellular flux analyzer, various parameters of mitochondrial bioenergetics during myocardium energy metabolism could be measured **(Supplementary Figure 3A**). Firstly, seeding cell density and FCCP concentration were optimized for the measurement of cellular metabolic functions. As shown in **Supplementary Figure 3B**, the optimal cell density of H9C2 cells was set at 5,000 cells/well, as to adjust an initial OCR to appropriate range (100–160 pmol/min) for further analysis. Meanwhile, the concentration of FCCP was optimized to 3 μM yielding maximal OCR (**Supplementary Figure 3B**). The concentration of oligomycin (1 μM) and rotenone/antimycin A (1 μM) were optimized according to the manufacturer’s instruction. The effects of increasing concentration of herbal extracts on OCR of cultured H9C2 cells were plotted against time. All extracts could dose-dependently increase basal respiration, proton leak, ATP production, and non-mitochondrial respiration to different degree (**Figure 2**). In mitochondrial bioenergetics, the effects of DBT, as well as other herbal extracts, to the parameters of maximum respiration and SRC were rather different. By analyzing the results of mitochondrial bioenergetics, all herbal extracts could regulate OCR reading in dose-dependent manners (**Figure 3**). DBT in many cases showed the best induction in basal respiration, proton leak, ATP production, and non-mitochondria respiration (**Figure 3**). The non-mitochondria respiration was the most sensitive parameter in responding to DBT challenge, i.e., ∼10-fold induction at high dose of DBT. In addition, the responsive dose of cultured cardiomyoblasts to herbal extracts was rather sensitive. In many cases, a low dose of herbal extract could achieve a maximal response (**Figure 3**).

**Figure 4:**
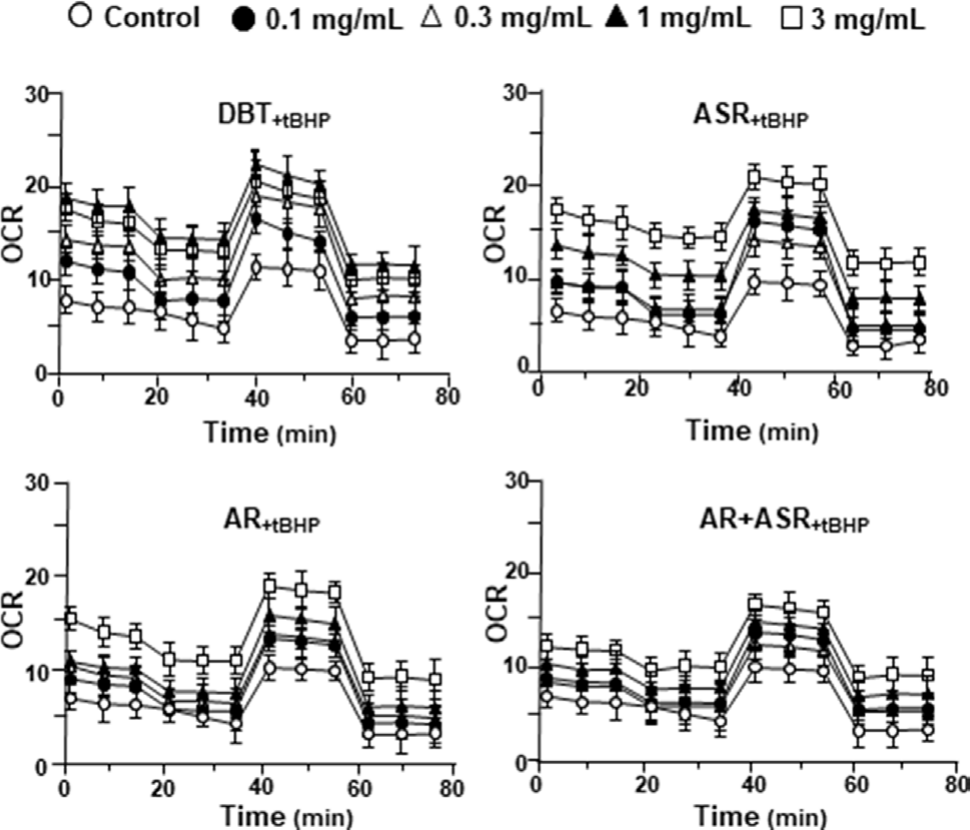
The extracts of DBT, ASR, AR, and ASR+AR modulate mitochondrial bioenergetics of H9C2 cells under oxidative stress. Cultured H9C2 cells were treated with herbal extracts for 24 h. Then, the treated cells were exposed to 30 µM tBHP for 3 h before measuring oxygen consumption rate with XFp Cell Mito Stress Test, as in **Figure 2**. The OCR value in pmol/min/mg of protein was shown. Blank is the naive cell. Data are expressed as mean ± SD, *n* = 3, each with triplicate samples.

**Figure 5:**
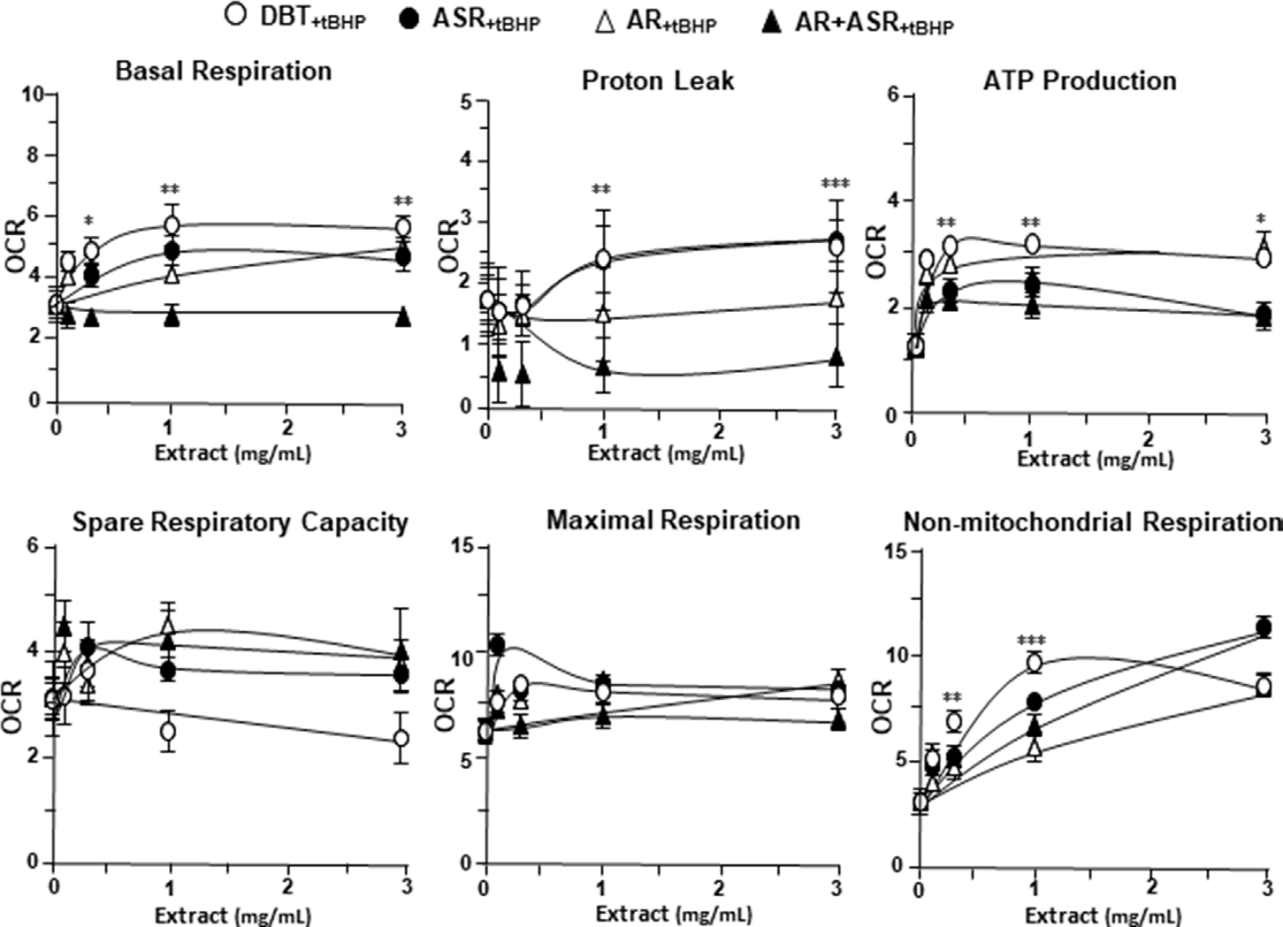
The parameters of mitochondrial respiration in DBT, ASR, AR, and ASR+AR applied tBHP-treated H9C2 cells. Cultured H9C2 cells were treated as that in **Figure 4**. The effects of increasing concentration of herbal extracts to basal respiration, proton leak, ATP production, spare respiratory capacity, maximal respiration, and non-mitochondrial respiration was measured and compared. The OCR value in pmol/min/mg of protein was shown. Data are expressed as mean ± SD, *n* = 3, each with triplicate samples. Statistical comparison was made of the sample with the lowest value of corresponding concentration, *p < 0.05, **p < 0.01, and ***p < 0.001.

The mitochondrial bioenergetics was carried out in tBHP-treated cardiomyoblasts under the treatments of herbal extracts (**Figure 4**). Similar to the control cell, the herbal extracts increased, dose-dependently, basal respiration, proton leak, ATP production, and non-mitochondrial respiration to different degree. The robust inductive effects in OCR reading after the treatment of DBT could be revealed in basal respiration, ATP production and non-mitochondrial respiration: these outcomes were very similar to that in normal cultured cardiomyoblasts (**Figure 5**). These results showed that the effects of DBT could be revealed in normal or stressed cell cultures. Again, all extracts showed a robust response in a low dose treatment (**Figure 5**).

**Figure 6:**
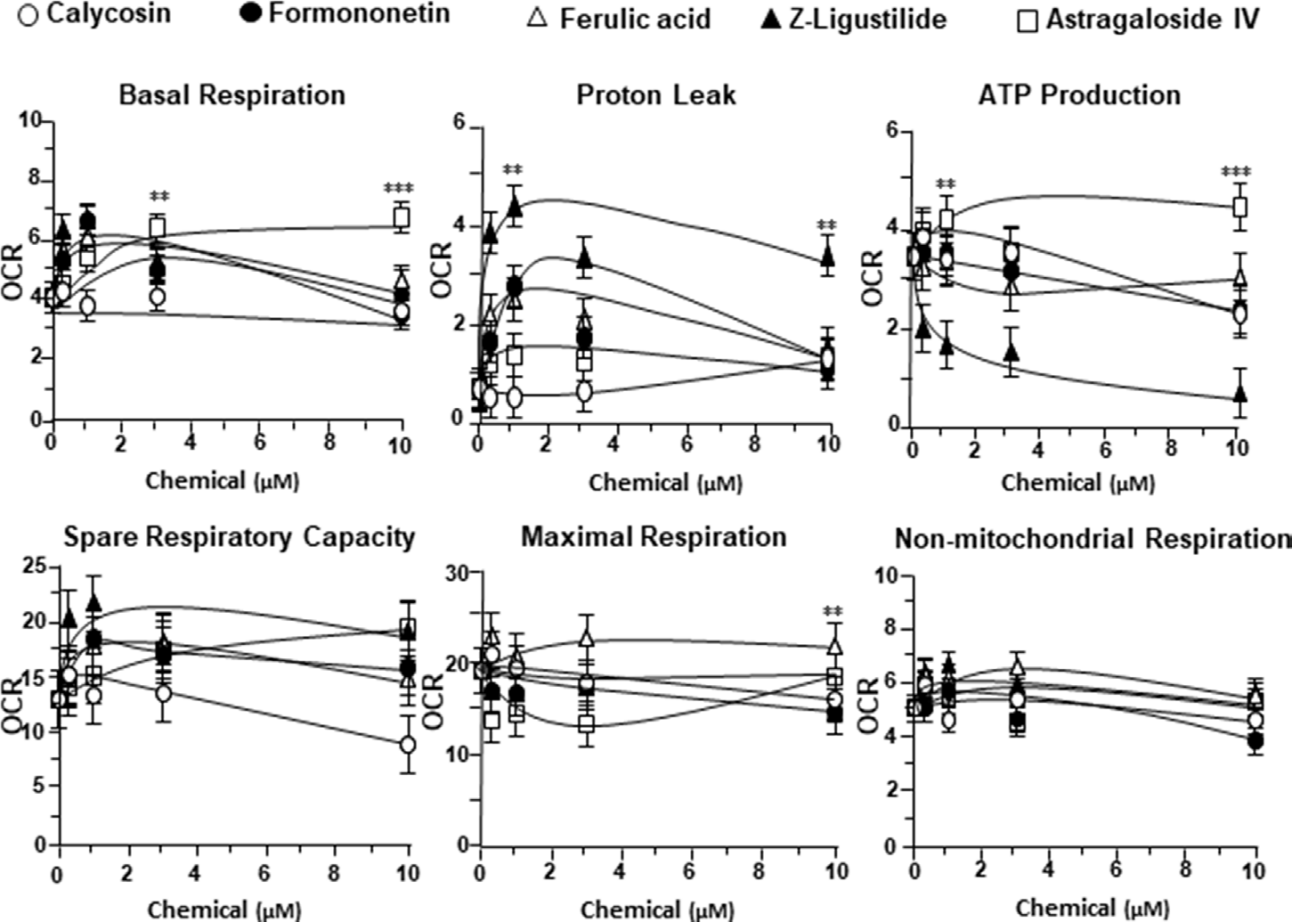
The parameters of mitochondrial respiration in tBHP-treated H9C2 cells in the present of major chemicals from DBT. The effects of increasing concentration of different extracts to basal respiration, proton leak, ATP production, spare respiratory capacity, maximal respiration, and non-mitochondrial respiration were measured and compared. The OCR value in pmol/min/mg of protein was shown. Data are expressed as mean ± SD, *n* = 3, each with triplicate samples. Statistical comparison was made with the sample with the lowest value of corresponding concentration, *p < 0.05, **p < 0.01, and ***p < 0.001.

To further investigate the bioenergetic roles of major ingredients in DBT, the mitochondrial bioenergetics was carried out in tBHP-treated cardiomyoblasts under the treatments of calycosin, formononetin, ferulic acid, Z-ligustilide, and astragaloside IV (**Figure S1A**). The robust inductive effects in OCR reading, after the treatment of astragaloside IV, could be revealed in basal respiration, ATP production, and SRC, but not for other DBT ingredients **(** **Figure 6**). Moreover, Z-ligustilide was able to induce proton leakage significantly (**Figure 6**).

### DBT Stabilizes Mitochondrial Membrane Potential

**Figure 7:**
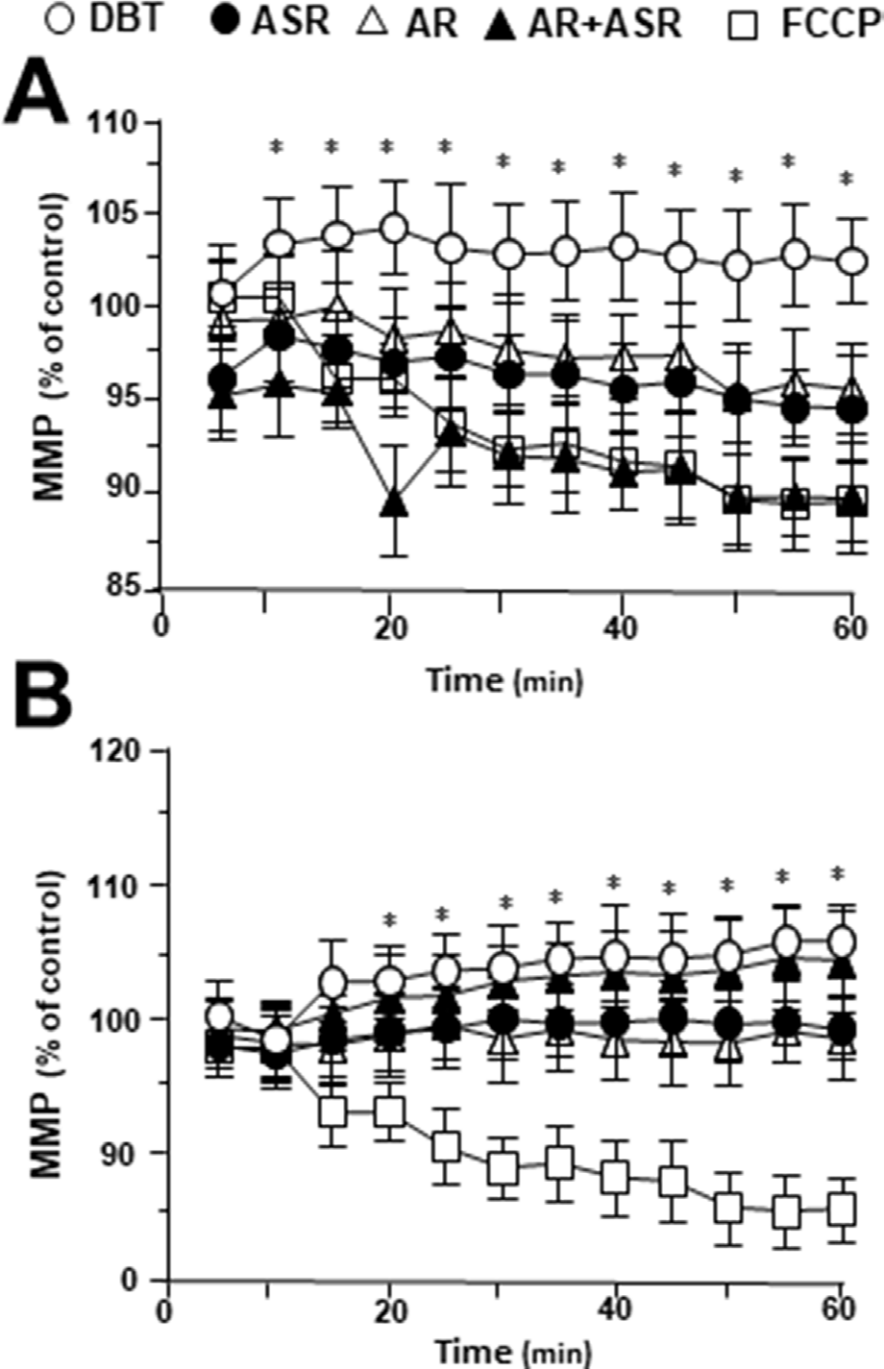
Regulation of mitochondrial membrane potential in H9C2 cells treated with herbal extracts. In a 96-well black multi-well plates with a clear bottom, H9C2 cells were stained with JC-1 and then washed with 2× PBS. The fluorescence of JC-1 aggregates in different treatments was measured. FCCP (100 µM) was used as a negative control. **(A)** Time course of herbal extract-induced changes in mitochondrial membrane potential in H9C2 cells. **(B)** Time course of herbal extract-induced changes in mitochondrial membrane potential in H9C2 cells after treated with 400 µM tBHP. All herbal extract was at 0.1 mg/ml. Data are expressed as the percentage of fluorescence intensity  of JC-1 aggregate to value of control (no treatment). Values are in mean ± SD, with *n* = 3. Statistical comparison was made with the sample with control, *p < 0.1, **p < 0.01, and ***p < 0.001.

To investigate mitochondrial membrane potential being regulated by DBT, the changes in the membrane potential were monitored by real-time photo-luminescent. The incubation of H9C2 cells with DBT induced an increase of mitochondrial membrane potential in a time-dependent manner. During initial time period (5–20 min), the membrane potential was induced by DBT application, and thereafter which was stabilized (**Figure 7A**). In contrast, a gradual decrease in mitochondrial membrane potential was observed after incubation with the extracts of AR, ASR, and AR + ASR. Furthermore, the effects of different herbal extracts on membrane potential in tBHP-treated H9C2 cells were tested. Interestingly, DBT applied in H9C2 cells caused a time-dependent manner increase of mitochondrial membrane potential during initial period (5–20 min): the maximum of stimulation being increased at 20 min after the herbal treatment (**Figure 7B**). However, the treatment of H9C2 with the extracts of ASR and AR caused a time-dependent stability in mitochondrial membrane potential. FCCP, a chemical uncoupler of mitochondria, served as a control to induce a gradual decrease in mitochondrial membrane potential in both types of cultures.

### DBT Upregulates Mitochondrial Gene Expressions

**Figure 8:**
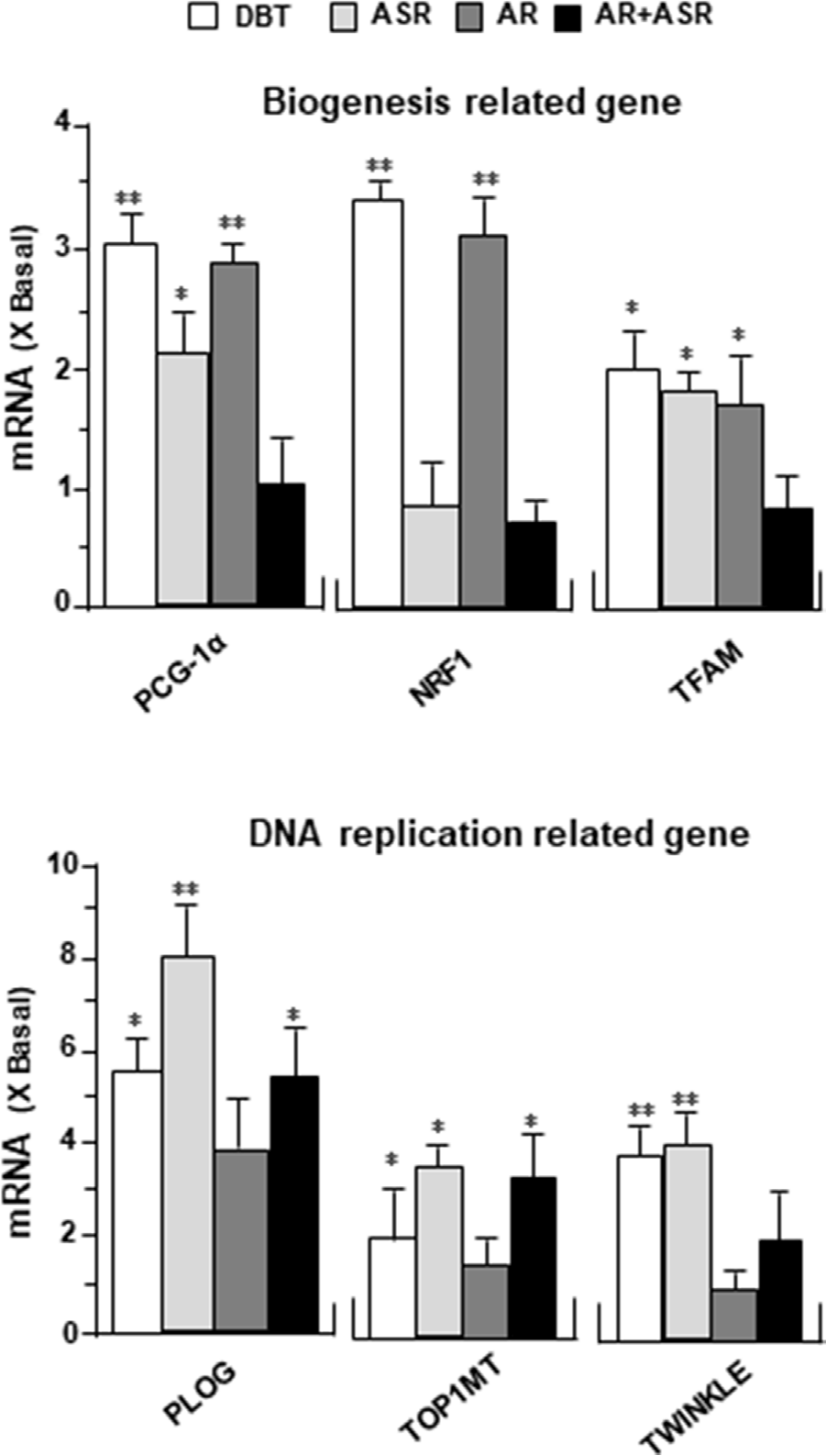
DBT regulates mitochondrial biogenesis-related genes. The expression levels of (upper panel) mitochondrial biogenesis related gene (PGC-1α, NRF1, TFAM) and (lower panel) mitochondrial DNA replication related gene (PLOG, TOP1MT, TWINKLE) were quantified by RT-PCR, following treatment of H9C2 cells with DBT, ASR, AR, and ASR+AR at 0.1 mg/ml for 24 h. Data are expressed as the ratio to the background (no treatment). Values are in mean ± SD, with *n* = 3. Statistical comparison was made of the sample with the background, *p < 0.1, **p< 0.01, and ***p < 0.001.

To determine the molecular mechanism responsible for enhanced mitochondrial content by DBT treatment, the expression levels of mitochondrial biogenesis related gene (PGC-1α, NRF1, TFAM) and mitochondrial DNA replication related gene (PLOG, TOP1MT, TWINKLE) were determined by quantitative real-time PCR. The treatments with DBT and AR extracts for 24 h caused a significant increase of PGC-1α mRNA to ∼3 folds (**Figure 8**). The PGC-1α mRNA induction was much less in cases of ASR and AR + ASR. In parallel, NRF1, a downstream target of PGC-1α, was also increased by ∼3 folds in DBT- and AR-treated cardiomyocytes. TFAM, a downstream effector of NRF1 and a regulator of mitochondrial DNA expression, showed a significant change of expression under DBT and ASR treatments (**Figure 8**). The mitochondrial DNA replication related genes, i.e. PLOG, TOP1MT, and TWINKLE, were measured here. DBT, as well as other herbal extracts, induced the expressions of these transcripts by two to eight folds (**Figure 8**).

### DBT Alters Mitochondrial Morphology

**Figure 9:**
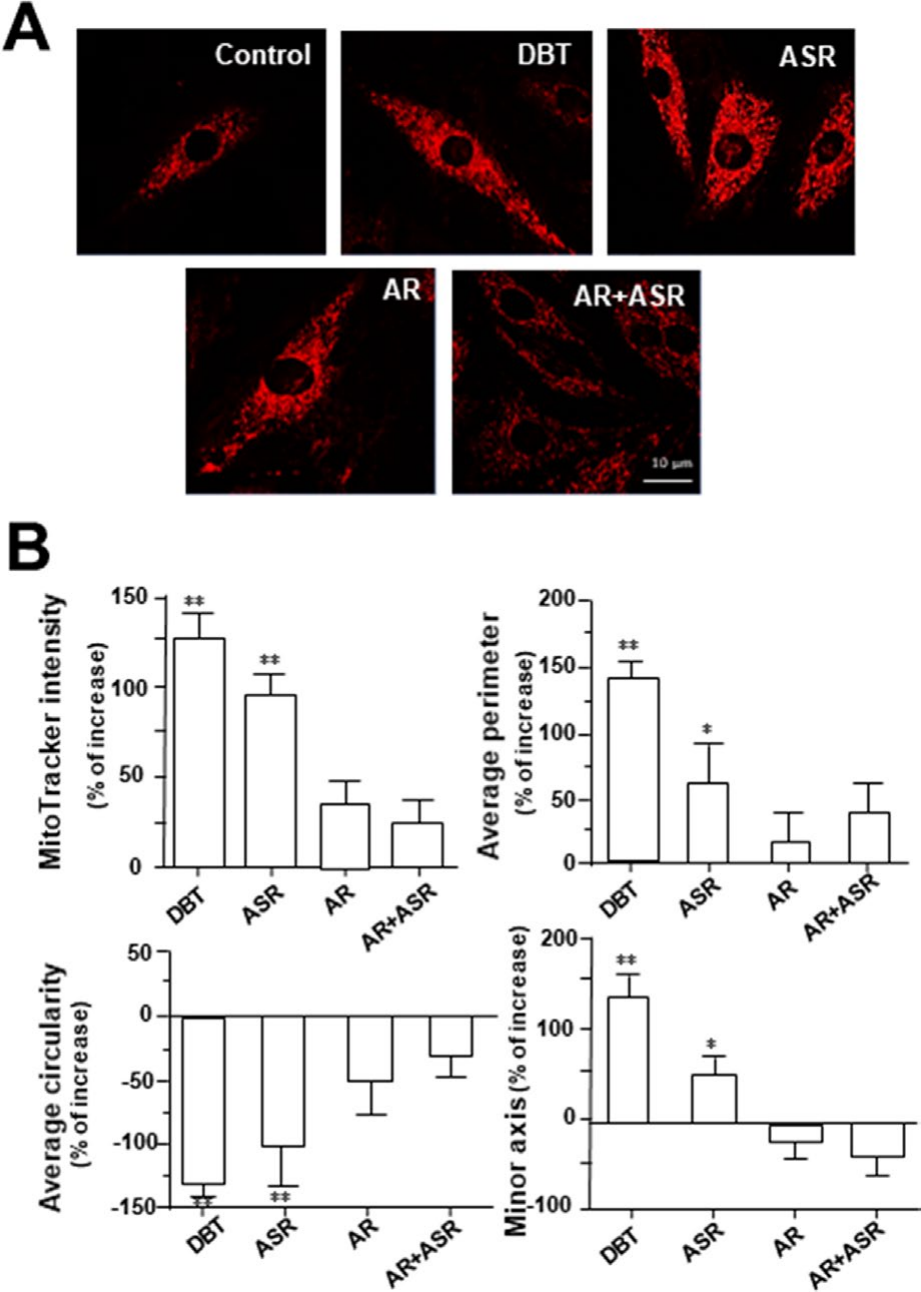
DBT increases mitochondrial morphology in cultured cardiomyocyte. **(A)** H9C2 cells were stained with MitoTracker Red FM and then analyzed for fluorescence intensity using confocal microscope. The cultures were treated with herbal extracts at 0.1 mg/ml for 24 h. Representing photo was shown. **(B)** Quantitative analysis from **(A)** out of 10 randomly selected images taken under 63× objective after treatment with DBT, ASR, AR, and AR+ASR. Data are expressed as the percentage of change to background (no treatment), in mean ± SD, with *n* = 4. Statistical comparison was made of the sample with control, *p < 0.1, **p < 0.01, and ***p < 0.001.

**Figure 10:**
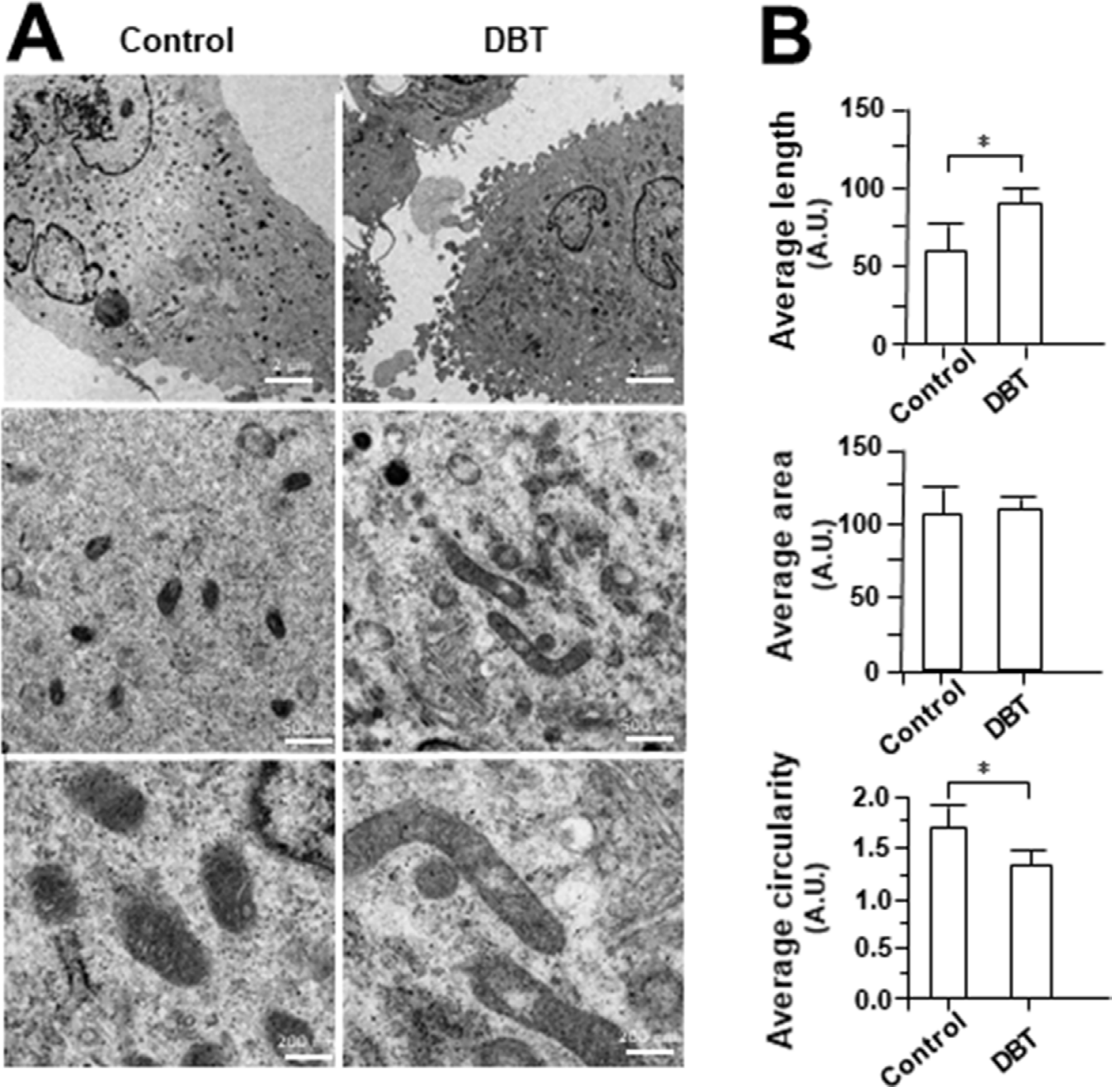
Electron microscope of DBT-treated H9C2 cells. **(A)** Mitochondrial ultrastructure was visualized by transmission electron microscope after H9C2 were treated with DBT (0.1 mg/ml) for 24 h. **(B)** Quantitative analysis from **(A)**, and data are expressed as arbitrary unit (AU), in mean ± SD, with *n* = 3. Statistical comparison was made of the sample with control, *p < 0.1.

MitoTracker staining and confocal microscopy were used to examine the oxidative status of mitochondria (**Figure 9A**). In DBT-treated cardiomyocytes, the MitoTracker staining was markedly increased by ∼160%, as compared to the control (**Figure 9B**). In addition, the mitochondria morphology of DBT-treated cells was changed, which included the average values of perimeter, circularity, and minor axis (**Figure 9B**). In addition, the mitochondria ultrastructure was revealed under a transmitted electron microscopy (**Figure 10A**). The mitochondria ultrastructure DBT-treated cardiomyocytes were found to have a decrease of circularity (**Figure 10B**). In contrast, the average length and area of mitochondria did not alter.

## Discussion

Mitochondria have crucial roles in cardiomyocytes’ dynamic, survival, and maintenance of cardiac functions in daily life (Gottlieb and Gustafsson, 2011). Mature cardiomyocytes possess a large number of mitochondria occupying at least 30% of total cell volume (Piquereau et al., 2013). The close relationship between mitochondrial biogenesis and cardiac function was supported by the observation of heart failure under mitochondrial dysfunction (Pisano et al., 2015). We have previously reported the protective effects of DBT in cardiomyocyte’s energy metabolism in mice having acute myocardial infarction or cardiac ischemia-reperfusion injury: this protective effect was triggered by mitochondrial enhancement as well as glutathione status in red blood cell (Mak et al., 2006). DBT is a Chinese herbal mixture being prescript for “qi-invigorating” action, and which is aiming to improve the “Yin-Yang balance” of an individual. The quantification of five major chemical ingredients, i.e., calycosin, astragaloside IV, formononetin, Z-ligustilide, and ferulic acid, in extracts of ASR, AR, and DBT were measured by using LC-MS. The result showed that DBT contained higher amount of these ingredients in decoction as compared to that of AR alone, ASR alone, or just AR+ASR (just mixing the extracts of AR and ASR together with boiling separately), and this result was in line to previous report (Zheng et al., 2014). The stability and solubility of bioactive ingredients in DBT could be increased after boiling of AR and ASR together in 5 to 1 ratio, in contrast to different herb ratios (Gao et al., 2016). On the other hand, the amount of bio-suppressive chemical (Z-ligustilide) was markedly reduced after boiling of two herbs together (Zheng et al., 2014). Based on the difference in contents of these compounds, the herbal extracts could be distinguished by an analysis of PCA, and the clustering of herbal extracts in PCA scoring plot was highly consistent with the chemical markers in loading plot. These results revealed that the chemical composition might be differed significantly in DBT, ASR, AR, and ASR+AR.

Due to flexibility and efficiency, Folin-Ciocalteu and DPPH radical scavenging assays were chosen here for chemical assessment of total antioxidant activities of herbal extracts (Kedare and Singh, 2011; Margraf et al., 2015). Here, the radical scavenging ability was represented relatively by total phenolic compounds. DBT had higher radical scavenging ability than that from the extracts deriving from single herb or ASR+AR mixture. In line with previous studies, DBT showed higher protection effects to oxidatively stressed cells, which therefore might be due to an inhibition of tBHP-induced ROS in the present of herbal extract (Gong et al., 2016b). In accordance to this notion, ferulic acid, derived from ASR, was demonstrated to be a key ingredient in orchestrating the anti-oxidative properties of DBT by reducing the formation of free radicals (Gong et al., 2016b).

Mitochondrial function plays roles in a variety of cellular process and metabolism. The assessment of bioenergetic properties in isolated mitochondria was done in past few years (Chowański et al., 2017). However, the damage of mitochondria during such isolation process happens very often, and additionally this isolation requires large amounts of starting materials (Lanza and Nair, 2009). To better understand the effect of DBT in mitochondrial metabolic flux change. The oxidative status of herbal extract-treated H9C2 cells was measured by an analyzer in monitoring real-time extracellular flux and mitochondria respiration in a live cell, which therefore could offer a more dynamic window for drug examination.

After DBT treatment in cultured H9C2 cells, an increase of basal respiration and ATP production was revealed. Astragaloside IV was identified to be a major active compound in inducing basal respiration and ATP production. Basal respiration is oxidative phosphorylation in responding to ATP demand; while ATP production is reflecting to cellular energy status, which can improve the energy metabolism in cultured cardiomyoblasts (Brand and Nicholls, 2011). On the other hand, the proton leak, a sign of mitochondrial uncoupling, was induced by DBT in our assay. Among different DBT ingredients, Z-ligustilide is a major inducer in proton leak. An increase of the proton leak is reported to reduce the generation of ROS (Dott et al., 2014) and to show the cytoprotective effect in ischemic injury models (Brookes, 2005). Indeed, the mitochondrial uncoupling has been proposed to have favorable effects in diseases having association with oxidative stress, e.g., ischemic-reperfusion injury, Parkinson’s disease, insulin resistance, aging, heart failure, ischemic-reperfusion injury, and obesity (Busiello et al., 2015). The non-mitochondria respiration, induced by DBT in the cultures, was having a dose-dependent increase; however, the induction was insignificant for DBT major chemicals. Thus, possible unidentified ingredients, e.g., polysaccharide, should be considered in triggering this non-mitochondria respiration. Besides the aforementioned bioenergetics parameters, we have shown the DBT-induced transcriptional expression of heme oxygenases *via* triggering hypoxia inducible factors (HIFs) (Dunn et al., 2014). Indeed, the effect of DBT on HIF pathway has been shown in previous report (Zheng et al., 2011). The protective effect of DBT under oxidative stress is in line to induction of heme oxygenases, because these enzymes are well known in affecting oxidation, differentiation, and apoptosis (Ayer et al., 2016). The detail mechanism therefore needs to further investigation.

In accounting the ATP production from oxidative phosphorylation, we have shown an increase of mitochondria membrane potential during DBT treatment. In our previous studies, astragaloside IV and calycosin are proposed to be active compounds in enhancing the stability of mitochondria membrane potential (Huang et al., 2018). Stability of mitochondria membrane potential is an important parameter in maintaining the mitochondrial function (Brand and Nicholls, 2011).

The energy lost in dissipating proton gradient *via* uncoupling protein (UCP) is the cause of heat generation, and therefore UCP is linking to thermogenesis (Dulloo and Samec, 2001). PGC-1α, a co-activator of PPARy, serves as an inducible booster in mitochondrial biogenesis (Wenz, 2013). Here, PGC-1α, as well as its down stream effectors including NRF1, TFAM, and other related genes involved in NRF family, was found to be upregulated in DBT-treated cardiomyocytes. PGC-1α is strongly related with fatty acid oxidation, glucose utilization, antioxidant detoxication, angiogenesis, mtDNA transcription, and replication (Liang and Ward, 2006). Furthermore, the transcriptional level of mtDNA polymerase genes (PLOG,TOP1MT, and TWINKLE) were also upregulated after DBT treatment. Report has been shown the important of mtDNA in aging process (Copeland, 2010).

The decrease of circularity and increasing of perimeter and minor axis of mitochondria could be reflected to cell having less oxidative stress (Sripathi et al., 2018). These mitochondria morphological parameters in cardiomyocytes could be altered by DBT treatment. In line to this result, astragaloside IV has been shown to improve mitochondrial ultrastructure of angiotensin II-induced dysfunction in vascular smooth muscle cells (Lu et al., 2015). In addition, ferulic acid applying together with ascorbic acid could restore the catecholamine-induced cardiotoxicity in rats (Yogeeta et al., 2006). Ligustilide, another key chemical in DBT, restored the memory deficiency in APP/PS1 transgenic mice by increasing the mitochondrial length (Xu et al., 2018). Therefore, we speculated that DBT treatment in cultured cardiomyoblast might promote mitochondrial biogenesis.

In summary, this study provided a comprehensive analysis of DBT effects on mitochondrial bioenergetics, as well as to explain the difference of tonic effect between single herbs and formulation of DBT. In our future work, the molecular docking has to be done in revealing the possible interaction of various molecular protein targets with DBT major ingredients: because the direct targets in mitochondria being activated by DBT are not known.

## Author Contributions

KT and KK conceived and designed the experiments. KK, YH, and LW performed experiments, analyzed the results, and made the figures and tables. YH and TD contributed to the scientific discussions. KT and KK wrote the paper.

## Funding

This study was supported by Hong Kong RGC Theme-based Research Scheme (T13-607/12R), Innovation and Technology Commission Innovation Technology Fund (UIM/288, UIM/302, UIM/340, UIT/137, ITS/022/16FP; ITCPD/17-9), TUYF15SC01, Shenzhen Science and Technology Committee Research Grant (CKFW2016082916015476; ZDSYS201707281432317; JCYJ20170413173747440; JCYJ20160229205812004; JCYJ20160229210027564), and Guangzhou Science and Technology Committee Research Grant (GZSTI16SC02; GZSTI17SC02).

## Conflict of Interest Statement

The authors declare that the research was conducted in the absence of any commercial or financial relationships that could be construed as a potential conflict of interest.

## Supplementary Material

Figure S1 PCA of chemical markers in different AR extracts. **(A)** Chemical Structure of calycosin, formononetin, ferulic acid, Z-ligustilide and astragaloside IV. **(B)** Identiﬁcation of ferulic acid (1), calycosin (2), astragaloside IV (3), Z-ligustilide (4) and formononetin (5) was made by an MS detector. Representative chromatograms of standard markers (Standards), DBT, ASR and AR under MRM mode were shown. **(C)** The scoring plot of different extracts was presented by comparing the contents of chosen standards. PC1 and PC2 described ∼80.2% and ∼17.5% of the total variability, respectively. **(D)** The loading plot of PC1 versus PC2 for four markers was shown, *n* = 3.

Figure S2
**(A)** Optimization of tBHP dose in MTT assay. Cultured H9C2 cells (1 x 104 cells/well) were exposed to tBHP at various concentrations. The cell viability was determined by MTT assay after treated for 3 hours, and the level of intracellular ROS was measured by fluorescent staining after 1 hour. Cell viability was expressed as % of control (cells without tBHP). tBHP at 400 µM was used for routine analysis. **(B)** The effects of DBT, ASR, AR and ASR+AR extracts and main ingredients for cell viability in H9C2 cells. Cultured H9C2 cells were treated with different extracts (0-3 mg/mL) for 24 hours. Cell viability was determined by MTT assay. Data are expressed as Mean ± SD, *n* = 3, each with triplicate samples.

Figure S3
**(A)** Schematic diagram of metabolic parameters of mitochondrial respiration measured by Seahorse Bioscience XFp extracellular flux analyzer. Basal respiration represents energetic demand of the cell under baseline conditions. Proton leak shows the remaining basal respiration and is the difference in OCR after oligomycin and rotenone/antimycin A (R&A) injection. ATP production is the difference between basal respiration and proton leak and represents the portion of basal respiration that is being used to drive ATP production. Maximal respiration shows the maximum rate of respiration that the cell can achieve, which is calculated as the OCR after FCCP injection. Spare respiratory capacity is the difference between maximal and basal OCR and can be an indicator of cell fitness or flexibility. The non-mitochondrial rate was subtracted from all other rates, which is a result of a subset of cellular enzymes that continue to consume oxygen after rotenone/antimycin A addition. **(B)** Optimization of cell density, FCCP and tBHP dosage in XFp Mito Stress Test. **(Left)** Cultured H9C2 cells with increasing cell density were seeded in XFp Cell Culture Miniplate and cultured for 48 hours before basal OCR was measured. **(Center)** H9C2 cells (5,000 cells/well) were cultured for 48 hours, then treated with 1 µM oligomycin and three serial injections of FCCP at different concentrations (a high concentration range of 3, 6, 12 µM and a low concentration range of 0.75, 1.5, 3 µM). The resulting data set characterizes the cells’ response to 6 doses of FCCP. **(Right)** Cultured H9C2 cells (5,000 cells/well) were exposed to tBHP at various concentrations for 24 hours, and OCR was determined. The above-mentioned OCR values were normalized with the cellular protein. Data are expressed as mean ± SD, *n* = 3, each with triplicate samples.

Table S1 Mass spectra properties of marker chemicals in DBT, ASR and AR extracts.
