# Supplementary figures and images for: Danggui Buxue Tang, a Chinese Herbal Decoction Containing Astragali Radix and Angelicae Sinensis Radix, Modulates Mitochondrial Bioenergetics in Cultured Cardiomyoblasts
Source: Front Pharmacol. 2019 Jun 21;10:614. doi: 10.3389/fphar.2019.00614 (PMC6611430; doi:10.3389/fphar.2019.00614)

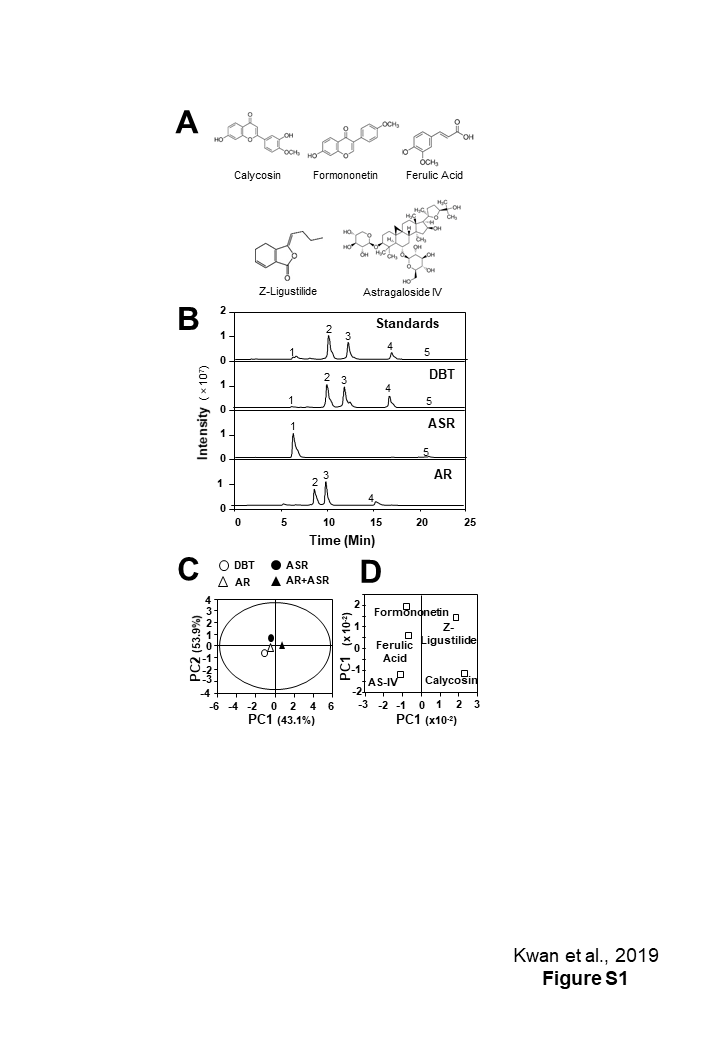

Supplement: Figure S1 — PCA of chemical markers in different AR extracts. (A) Chemical Structure of calycosin, formononetin, ferulic acid, Z-ligustilide and astragaloside IV. (B) Identiﬁcation of ferulic acid (1), calycosin (2), astragaloside IV (3), Z-ligustilide (4) and formononetin (5) was made by an MS detector. Representative chromatograms of standard markers (Standards), DBT, ASR and AR under MRM mode were shown. (C) The scoring plot of different extracts was presented by comparing the contents of chosen standards. PC1 and PC2 described ∼80.2% and ∼17.5% of the total variability, respectively. (D) The loading plot of PC1 versus PC2 for four markers was shown, n = 3. [file Image_1.tif]

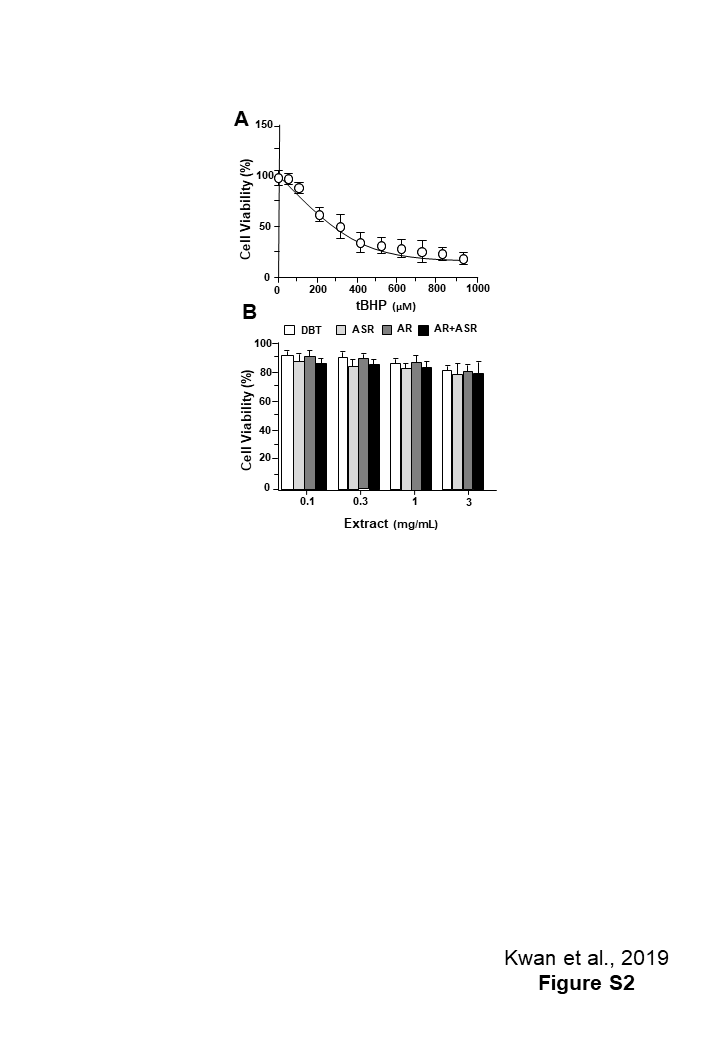

Supplement: Figure S2 — (A) Optimization of tBHP dose in MTT assay. Cultured H9C2 cells (1 x 10^4^ cells/well) were exposed to tBHP at various concentrations. The cell viability was determined by MTT assay after treated for 3 hours, and the level of intracellular ROS was measured by fluorescent staining after 1 hour. Cell viability was expressed as % of control (cells without tBHP). tBHP at 400 µM was used for routine analysis. (B) The effects of DBT, ASR, AR and ASR+AR extracts and main ingredients for cell viability in H9C2 cells. Cultured H9C2 cells were treated with different extracts (0-3 mg/mL) for 24 hours. Cell viability was determined by MTT assay. Data are expressed as Mean ± SD, n = 3, each with triplicate samples. [file Image_2.tif]

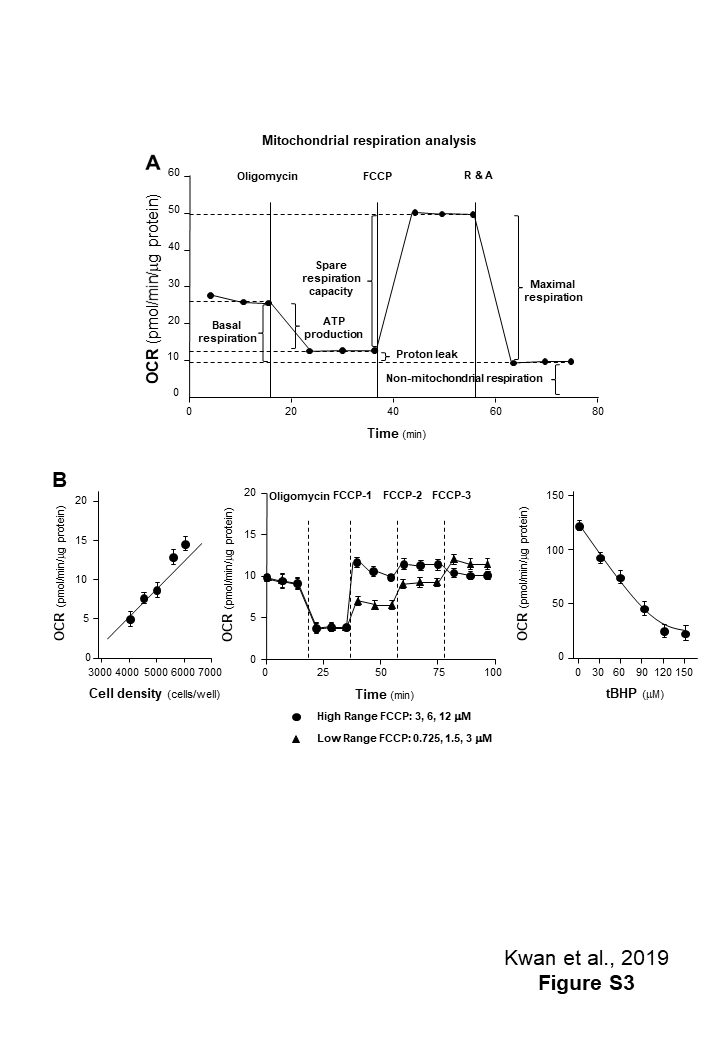

Supplement: Figure S3 — (A) Schematic diagram of metabolic parameters of mitochondrial respiration measured by Seahorse Bioscience XFp extracellular flux analyzer. Basal respiration represents energetic demand of the cell under baseline conditions. Proton leak shows the remaining basal respiration and is the difference in OCR after oligomycin and rotenone/antimycin A (R&A) injection. ATP production is the difference between basal respiration and proton leak and represents the portion of basal respiration that is being used to drive ATP production. Maximal respiration shows the maximum rate of respiration that the cell can achieve, which is calculated as the OCR after FCCP injection. Spare respiratory capacity is the difference between maximal and basal OCR and can be an indicator of cell fitness or flexibility. The non-mitochondrial rate was subtracted from all other rates, which is a result of a subset of cellular enzymes that continue to consume oxygen after rotenone/antimycin A addition. (B) Optimization of cell density, FCCP and tBHP dosage in XFp Mito Stress Test. (Left) Cultured H9C2 cells with increasing cell density were seeded in XFp Cell Culture Miniplate and cultured for 48 hours before basal OCR was measured. (Center) H9C2 cells (5,000 cells/well) were cultured for 48 hours, then treated with 1 µM oligomycin and three serial injections of FCCP at different concentrations (a high concentration range of 3, 6, 12 µM and a low concentration range of 0.75, 1.5, 3 µM). The resulting data set characterizes the cells’ response to 6 doses of FCCP. (Right) Cultured H9C2 cells (5,000 cells/well) were exposed to tBHP at various concentrations for 24 hours, and OCR was determined. The above-mentioned OCR values were normalized with the cellular protein. Data are expressed as mean ± SD, n = 3, each with triplicate samples. [file Image_3.tif]
