# Supplementary material for: Danggui Buxue Tang, a Chinese Herbal Decoction Containing Astragali Radix and Angelicae Sinensis Radix, Modulates Mitochondrial Bioenergetics in Cultured Cardiomyoblasts
Source: Front Pharmacol. 2019 Jun 21;10:614. doi: 10.3389/fphar.2019.00614 (PMC6611430; doi:10.3389/fphar.2019.00614)
Supplement: Table S1 — Mass spectra properties of marker chemicals in DBT, ASR and AR extracts. [file Table_1.docx]

**Table S1**: Mass spectra properties of marker chemicals in DBT, ASR and AR extracts.

| Chemical | Formula | Calculated mass [M] | Precursor ion [M-H]^a)^ | Fragmentor energy^b^ | | Collison energy^c^ | Product ion^d)^ |
| --- | --- | --- | --- | --- | --- | --- | --- |
| Calycosin | C_16_H_12_O_5_ | 284.1 | 283.1 | 100 | 13 | | 268 |
|  |  |  |  |  | 29 | | 211 |
| Astragaloside IV | C_41_H_68_O_14_ | 784.9 | 829.5^e)^ | 190 | 5 | | 829.5 |
|  |  |  |  |  | 25 | | 783.2 |
| Ferulic acid | C_10_H_10_O_4_ | 194.1 | 193.1 | 100 | 9 | | 134 |
|  |  |  |  |  | 9 | | 178 |
| Formononetin | C_16_H_12_O_4_ | 268.1 | 267.1 | 150 | 17 | | 252 |
|  |  |  |  |  | 29 | | 223 |
| Z-ligustilide^f)^ | C_16_H_12_O_4_ | 190.1 | 191.1 | 100 | 24 | | 91 |
|  |  |  |  |  | 49 | | 77 |
| Ginsenoside Rg_1_ | C_42_H_72_O_24_ | 800.5 | 799.5 | 250 | 5 | | 799.5 |
|  |  |  |  |  | 21 | | 637.3 |

^a)^The detected chemicals had better responses under the negative mode: the [M-H]^-^ was used as the precursor ion.

^b)^The fragmentor energy was optimized to have the greatest ionize efficiency.

^c)^The collision energy was optimized to have the greatest product ion intensity, which was the key factor in the MRM mode.

^d)^Two pairs of collision energy and product ions were used for the MRM analysis to guarantee the precision of analytes. The upper one was used for quantitative analysis and the lower own was for qualitative analysis.

^e)^The precursor ion of astragaloside IV was [M + HCOOH - H]^-^ under the negative mode.

^f)^Z-ligustilide had greatest response to the positive mode: the [M+H]^+^ was used as the precursor ion.
